# King Edward's Fund Policy

**Published:** 1921-04-23

**Authors:** 


### The Hospital

The Workers' Newspaper of Administrative Medicine and Institutional Life. Administration. National Insurance and Health.

No. 1820, Vol. LXX. SATURDAY, APRIL 23, 1921. PRICE TWOPENCE

### KING EDWARD'S FUND POLICY.

Following on the famous thirteen resolutions dealing with the policy to be recommended for the preservation of the voluntary system, the King's Fund Policy Committee has issued an interim report, making a statement of the situation (p. 67) and its views thereon. It will be remembered that, roughly speaking, the problem of the financial position last year was solved by the distribution of an emergency grant from the Fund and by grants made from the National Relief Fund. This, of course, only applies to a collective view of the hospitals of London together; naturally, some were better and some were worse off. But the chief point is that these grants are not recurrent.

We now come, however, to the problem of 1921. As the Committee points out, it is impossible to consider a further emergency grant from the Fund, and the National Relief Fund has been closed. Naturally, some of the hospitals, especially large ones, are in a bad way, and the various expedients of increasing their income, even if successful, cannot retrieve the position this year, however much they may build up strength for the future. The Policy Committee hints at two solutions. One is a Government grant in view of what is still owing on account of the accommodation of Service men during the war. This debt is chiefly in respect of the free use of the capital (*i.e.*, building and equipment) of the voluntary hospitals, no account of which was ever taken in estimating the capitation grant. Another possible solution is a lump grant from the accumulated funds of the Approved Societies. With the first of these we have always been in accord; and we believe that we were the first to call attention to the fallacy of the capitation grant, as it has been calculated in the past. However, any Government grant can only refer to hospitals which received soldiers and sailors. They were, of course, but a certain proportion of the hospitals generally.

With regard to the second suggestion, we cannot so readily concur, and think that it would be far better for the Approved Societies to fund substantial sums in order to finance an immediate scheme of payment for services rendered to their members whilst in or attending hospitals.

A feature of the report which will not be received with entire concurrence by the hospitals is that which would suggest the extension of the powers of the King's Fund as a disciplinary body. The Policy Committee is most modest on this point, but, all the same, some would like to see, when proposals are brought forward of this nature, some suggestion that the hospitals will be better represented, possibly through an electoral scheme, upon the General Council of the Fund.

